# A cross-sectional study of the identification of prevalent asthma and chronic obstructive pulmonary disease among initiators of long-acting β-agonists in health insurance claims data

**DOI:** 10.1186/1471-2466-14-47

**Published:** 2014-03-19

**Authors:** David D Dore, Najat Ziyadeh, Bin Cai, C Robin Clifford, Heather Norman, John D Seeger

**Affiliations:** 1 Departments of Health Services, Policy & Practice and Epidemiology, Brown University School of Public Health, Brown University, Box G-121-7, 121 South Main Street, Providence, RI 02903, USA; 2 Center for Gerontology and Health Care Research, Brown University School of Public Health, Providence, RI, USA; 3 Optum Epidemiology, Waltham, MA, USA; 4 Novartis Pharmaceuticals, East Hanover, NJ, USA; 5 Division of Pharmacoepidemiology and Pharmacoeconomics, Brigham and Women’s Hospital/Harvard Medical School, Boston, MA, USA

**Keywords:** Claims data, Risk management, Off-label prescribing, Asthma, Chronic, Obstructive pulmonary disease, Long-acting beta agonis

## Abstract

**Background:**

Claims data are potentially useful for identifying long-acting β-agonist (LABA) use by patients with asthma, a practice that is associated with increased mortality. We evaluated the accuracy of claims data for classifying prevalent asthma and chronic obstructive pulmonary disease (COPD) among initiators of LABAs.

**Methods:**

This study included adult LABA initiators during 2005–2008 in a US commercial health plan. Diagnosis codes from the 6 months before LABA initiation identified potential asthma or COPD and a physician adjudicated case status using abstracted medical records. We estimated the positive predictive value (PPV) and 95% confidence intervals (CI) of covariate patterns for identifying asthma and COPD.

**Results:**

We sought 520 medical records at random from 225,079 LABA initiators and received 370 (71%). The PPV for at least one asthma claim was 74% (CI 63–82), and decreased as age increased. Having at least one COPD claim resulted in a PPV of 82% (CI 72–89), and of over 90% among older patients, men, and recipients of inhaled anticholinergic drugs. Only 2% (CI 0.2–7.6) of patients with a claim for COPD alone were found to have both COPD and asthma, while 9% (CI 4–16) had asthma only. Twenty-one percent (CI 14–30) of patients with claims for both diagnoses had both conditions. Among patients with no asthma or COPD claims, 62% (CI 50–72) had no confirmed diagnosis and 29% (CI 19–39) had confirmed asthma.

**Conclusions:**

Subsets of patients with asthma, COPD, and both conditions can be identified and differentiated using claims data, although categorization of the remaining patients is infeasible. Safety surveillance for off-label use of LABAs must account for this limitation.

## Background

Although long-acting β-agonist (LABA) medications improve lung function in asthma and chronic obstructive pulmonary disease (COPD) by inducing relaxation of bronchial smooth muscle and inhibiting release of mediators of hypersensitivity [1], their use in asthma, at least in the absence of concomitant inhaled corticosteroids (ICS), has been associated with more asthma-related deaths [2]. These findings, though not uniform [3], have led to increased regulatory restrictions on the use of LABA, including the implementation of Risk Evaluation and Mitigation Strategies (REMS) that aim to inform prescribers and patients about these risks [4]. Indeed, some LABAs have only an indication for COPD [5].

Monitoring whether patients with asthma only use LABAs has the potential to ensure that prescribers are using the products in accordance with known safety data. Physicians should generally avoid prescribing LABA as monotherapy for asthma (an “off-label” use), so that unnecessary harms in patients with asthma are avoided, while ensuring that the product reaches patients with COPD (an “on-label” use), in whom LABA risk-benefit profiles appear acceptable. Health insurance claims data provide an efficient core for this type of surveillance, but the data must accurately characterize LABA users’ likely indication for use of the drugs (asthma or COPD).

Indeed, COPD and asthma are distinct clinical entities, but share symptoms and treatments. COPD is characterized by non-reversible airflow restriction or obstruction due to chronic bronchitis or emphysema [6]. Asthma involves recurrent airway obstruction, hyper-responsiveness, and inflammation [7]. First onset of asthma generally occurs at a young age, while first onset of COPD typically occurs among patients over 40 years of age.

The similarities in these conditions complicate differentiation of COPD and asthma in claims data. Therefore the objective of this study was to identify patterns of health insurance claims for asthma and COPD among users of LABA (with or without ICS use) that might improve the categorization of individual patients with asthma or COPD. We studied only new users of LABA to quantify the measurement characteristics of the data for identifying the indication for treatment so that these data would directly inform safety surveillance for LABA.

## Methods

### Data sources

The study population came from the Normative Health Information Database, a claims database of a large US commercial health plan (UnitedHealth Care). Diagnoses associated with the claims are recorded using the International Classification of Disease, 9th revision (ICD-9) coding system. Procedures map to ICD-9, Common Procedural Terminology (CPT), and the Centers for Medicare and Medicaid Services Common Procedure Coding System (HCPCS) codes. National Drug Codes (NDCs) identify medications.

### Cohort formation

**Figure 1:**
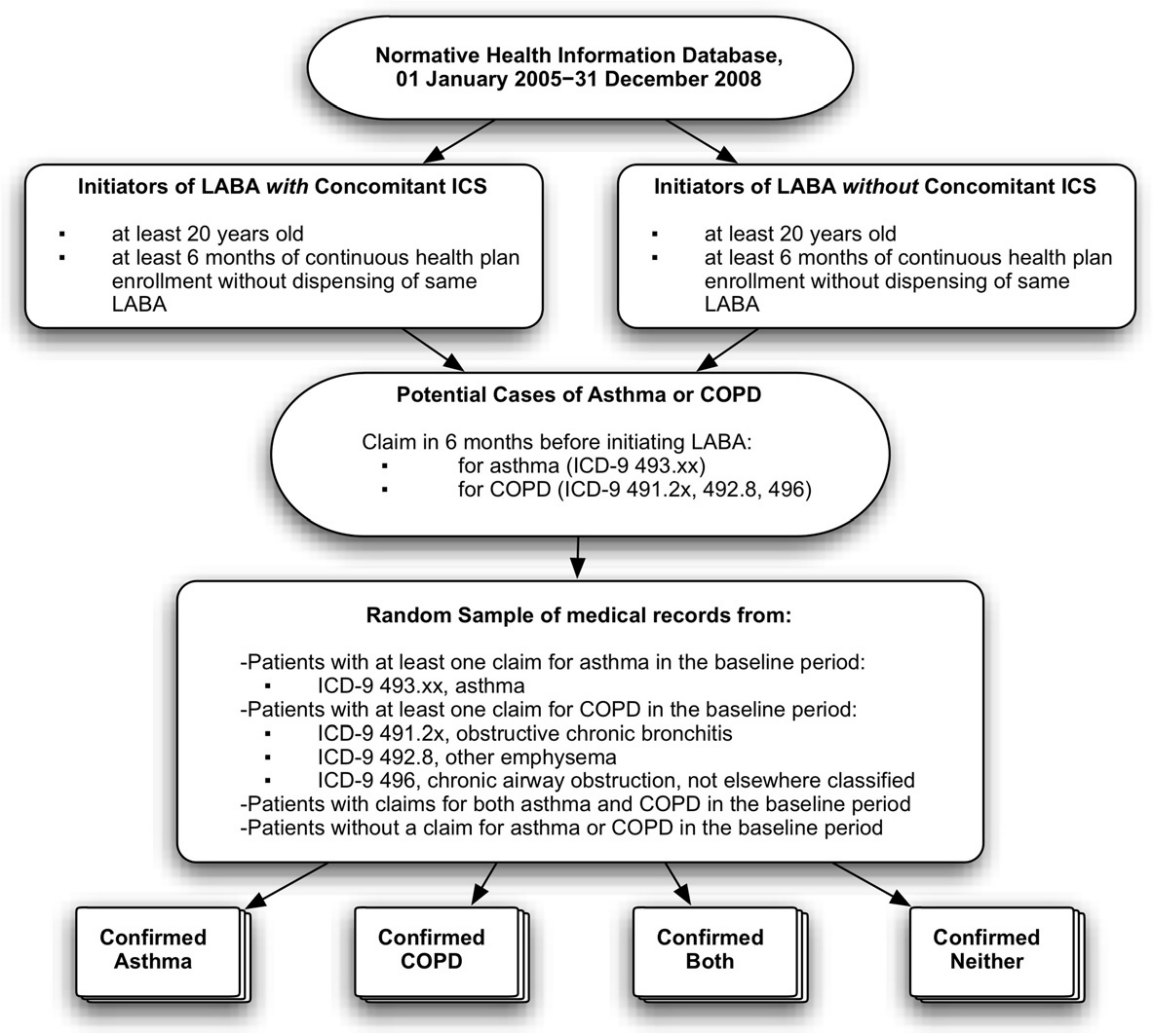
**Formation of study cohorts from the Normative Health Information, and Identification and Confirmation of Prevalent Asthma and COPD, 1/1/2005–12/31/2008.** LABA, identifying long-acting β-agonist; ICS, inhaled corticosteroid; COPD, chronic obstructive pulmonary disease; ICD, International Classification of Diseases.

Eligible patients were initiators or switchers of LABA or LABA and ICS combinations who were at least 20 years old between 01 January 2005 and 31 December 2008 (Figure 1). We included all LABA and ICS products on the U.S. market during the study period. The LABAs were: arformoterol, formoterol, and salmeterol. The ICS were: beclomethasone, budesonide, ciclesonide, flunisolide, fluticasone, mometasone, and triamcinolone.

We defined initiation as a dispensing of a LABA preceded by 6 months of continuous enrollment without a dispensing of the same LABA. Switchers were patients who initiated a new LABA, but used another LABA during the previous 6 months. The study began for each patient at their first eligible LABA dispensing (index date). The 6 months prior to the index date was the period from which we identified diagnosis, procedure, and medication codes that characterized the study population. Patients were considered concomitant users of LABA and ICS upon initiation of a combination LABA/ICS product or if they initiated a LABA and received a dispensing of an ICS on the same day.

Patients with a recent previous dispensing of an ICS were in the monotherapy group. While requiring that patients received the combination formulation of LABA/ICS or concomitant ICS on the same day as the initial LABA dispensing may result in misclassification of individuals who received ICS and LABA on different days, this more stringent definition increases the likelihood that we include only users concomitant therapy in the combination LABA/ICS group. This preference is important because combination LABA/ICS for asthma appears safer than LABA monotherapy and should not be discouraged. (Empirically, this decision was unimportant. Ninety-six percent of patients initiated on a combination product. Of the 9,965 patients classified as LABA-only initiators, 1,931 [19%] used ICS in the baseline period and were potentially misclassified. This number represents only 0.9% of the full study population, and this misclassification is of little consequence).

### Identification of asthma and COPD

Potential cases of asthma or COPD had ICD-9 diagnosis codes for asthma or COPD in any diagnosis field on claims occurring in the 6 months prior to the index date on an inpatient or outpatient claim (a similar approach requiring 12 months of continuous enrollment yielded similar results). Patients with at least one code for asthma, COPD, both, or neither in the baseline period were eligible for subsequent medical record abstraction. We excluded from consideration certain diagnosis codes that might include COPD, but are mixed with other disorders, including ICD-9 490 (bronchitis, not specified as acute or chronic) [6]. Because hospitalization for asthma is rare relative to the prevalence of the disease, we did not distinguish between inpatient and outpatient claims. We chose to develop our own definition of asthma and COPD despite the existence of algorithms in the literature (e.g., Mapel et al. [8] and Dombkowski et al. [9]), because in our work we aimed to classify baseline diagnoses (indications), whereas most definitions in the literature apply specifically to outcomes or cohort definitions. Definitions of outcomes and covariates are more robust to insensitive measures of disease than case criteria for identification of off-label use.

To verify the diagnoses, we sought a random sample of 130 medical records (with the goal of obtaining 100) from each of the following 4 categories of eligible patients (520 patients in total):

Patients with at least one claim for asthma:

ICD-9 493.xx, asthma

Patients with at least one claim for COPD:

ICD-9 491.2x, obstructive chronic bronchitis

ICD-9 492.8, other emphysema

ICD-9 496, chronic airway obstruction, not elsewhere classified

Patients with claims for both asthma and COPD

Patients without a claim for either asthma or COPD

We sought a total of 640 records, including alternate records for 120 of the 520 sampled patients. We sought medical records from hospitals and physicians’ offices using a 2-step process. First, we created a chronological listing of insurance claims for the sampled patients noting the date of service corresponding to the diagnosis code of interest (asthma or COPD). Patients with claims for both diagnoses had the one closest to the LABA initiation date noted, and those with neither had no claim noted.

These claims listings were reviewed to identify the provider or facility from which the medical record was likely to document the diagnosis. Trained abstractors contacted the provider or facility seeking the medical record. For records with clearly inadequate information (as assessed prior to the adjudication process), we re-contacted providers to request additional information. If additional information was not obtained for these cases, the charts were not adjudicated and did not enter analysis. Incomplete medical records were those without clinical information available (i.e., the contained administrative material only). Records with clinical information, even if incomplete, were forwarded to the adjudicators for review.

A physician external to the study team, blinded to study medications, adjudicated the presence of asthma and COPD using the Global Initiative for Chronic Obstructive Lung Disease (GOLD) criteria for COPD [10] and the third Expert Panel Report of Guidelines on Asthma [11]. Confirmation required the affirmative listing of the case criteria in the medical records. The Additional file 1 includes additional information on the case adjudication process.

### Analysis

We initially tabulated characteristics of the study population from the 6 months before LABA initiation, including whether LABA initiators were apparent naïve users of all LABAs, the prevalence of comorbidities, and the intensity of healthcare utilization characteristics, such as outpatient visits, emergency department visits, hospitalizations, and home oxygen use. Next, we empirically identified covariates by listing the 100 most prevalent (top 100) drug classes, top 100 diagnoses (at the 3-digit ICD-9 level) and the top 100 procedures received by LABA users, stratifying by ICS.

Using adjudication results as the gold standard, we then estimated the association between the baseline characteristics and the adjudicated diagnosis, with the aim to distinguish between patients with confirmed asthma and those without, and those with confirmed COPD and those without.

We estimated the positive predictive value (PPV) of claims identification of asthma or COPD stratified by the 3 covariates with the largest absolute difference in prevalence across patients with confirmed and unconfirmed asthma. We use this approach among all patients except for those with only a claim for COPD. Whereas for patients with claims for asthma or no claim for asthma or COPD, we were interested in the fraction with asthma (and therefore contraindications to LABA therapy), for patients with claims-based COPD only, we were interested in the fraction with true COPD (i.e., an appropriate indication for LABA therapy). For the latter set, we compared categories of confirmed COPD vs. unconfirmed COPD to identify the 3 most individually discriminating covariates. We estimated 95% confidence intervals (CI) using the exact binomial method.

We chose this categorization of asthma and COPD to be useful for monitoring off-label prescribing of LABAs, where it is of interest to know what fraction of patients with claims for COPD truly have COPD (on-label use—the latter set), and for patients with at least one claim for asthma or for patients with no claim for asthma or COPD, what fraction truly have asthma only (off-label use—the former set). These analyses were restricted to patients for whom we received a medical record. Additionally, we tabulated these PPVs within categories of a number of clinically derived variables that could plausibly be associated with a different PPV and within covariate patterns defined by the 3 most individually discriminating covariates.

We also conducted a *post-hoc* regression analysis as an additional means to identify covariates whose presence may modify the accuracy of the identification of asthma or COPD. Using multinomial logistic regression models, we regressed confirmed case status (asthma only, COPD only, both, or neither, with neither as the referent) on the claims definitions for these conditions, plus the covariates with product terms for each covariate and the claims definitions. We retained an empirical approach to covariate selection by using a stepwise algorithm, entering and retaining variables with p-values < 0.05.

### Human subjects considerations

Since this study used protected health information (PHI) to link insurance claims to patient’s medical records, we operated with the oversight of the New England Institutional Review Board who approved our protocol, privacy practices, and whose Privacy Board granted a waiver of authorization for the use of PHI without obtaining patient consent.

## Results

Of the 225,079 eligible initiators of a LABA, 9,965 (4.4%) appeared to receive LABA monotherapy (Table 1). Users of a LABA without concomitant ICS were more likely to be ≥ 65 years of age, while there were no observed differences with respect to sex. Women accounted for nearly two-thirds of patients with baseline claims for asthma, while men accounted for over one-half of patients with COPD. A total of 107,839 of the 225,079 (47.9%) patients had no claim for asthma or COPD in the baseline period. The distribution of number of diagnoses of asthma or COPD in the baseline period was low, ranging from a mean of 0.6 in the 0–90 days before the index date to 0.18 in the 91–183 days before the index date.

**Table 1:** Select baseline demographic and clinical characteristics of initiators of long-acting β-agonist and patients with confirmed asthma or COPD status, January 1, 2005 – December 31, 2008

	**LABA**		**LABA & ICS**		**Asthma**		**COPD**		**Asthma & COPD**		**Neither Asthma nor COPD**	
	**(N = 9,965)**		**(N = 215,114)**		**(N = 87,864)**		**(N = 20,934)**		**(N = 8,442)**		**(N = 107,839)**	
	**N**	**%**	**N**	**%**	**N**	**%**	**N**	**%**	**N**	**%**	**N**	**%**
Age												
20-39	2,081	20.9	65,018	30.2	33,717	38.4	758	3.6	673	8.0	31,951	29.6
40-64	6,230	62.5	130,968	60.9	50,206	57.1	13,775	65.8	5,895	69.8	67,322	62.4
>64	1,654	16.6	19,128	8.9	3,941	4.5	6,401	30.6	1,874	22.2	8,566	7.9
Women	5,962	59.8	131,390	61.1	56,437	64.2	9,913	47.4	5,103	60.5	65,899	61.1
*Upper respiratory tract conditions*												
Sinusitis	1,812	18.2	42,469	19.7	20,310	23.1	2,828	13.5	1,794	21.3	19,349	17.9
Rhinitis	2,356	23.6	50,513	23.5	32,031	36.5	2,228	10.6	1,965	23.3	16,645	15.4
Tonsillitis	61	0.6	1,596	0.7	781	0.9	60	0.3	38	0.5	778	0.7
Nasopharyngitis and Pharyngitis	664	6.7	16,636	7.7	7,393	8.4	859	4.1	597	7.1	8,451	7.8
Laryngitis	109	1.1	2,062	1.0	797	0.9	197	0.9	125	1.5	1,052	1.0
*Lower respiratory tract conditions*												
Asthma	4,095	41.1	92,211	42.9	87,864	100.0	0	0.0	8,442	100.0	0	0.0
COPD	2,428	24.4	26,948	12.5	0	0.0	20,934	100.0	8,442	100.0	0	0.0
Emphysema	593	6.0	4,633	2.2	230	0.3	3,452	16.5	1,159	13.7	385	0.4
Bronchitis/Bronchiolitis	3,096	31.1	74,688	34.7	25,329	28.8	11,954	57.1	5,745	68.1	34,756	32.2
Bronchiectasis	154	1.5	1,116	0.5	300	0.3	351	1.7	170	2.0	449	0.4
Pneumonia (viral or bacterial)	911	9.1	15,371	7.1	4,629	5.3	3,948	18.9	1,954	23.1	5,751	5.3
*Other respiratory disease*												
Pleurisy	331	3.3	4,022	1.9	919	1.0	1,580	7.5	555	6.6	1,299	1.2
Pneumothorax	58	0.6	588	0.3	98	0.1	313	1.5	87	1.0	148	0.1
Respiratory failure	275	2.8	2,775	1.3	432	0.5	1,445	6.9	732	8.7	441	0.4
Pulmonary embolism	124	1.2	1,692	0.8	459	0.5	592	2.8	248	2.9	517	0.5
Pulmonary edema	154	1.5	2,411	1.1	629	0.7	780	3.7	344	4.1	812	0.8
Congestive heart failure	487	4.9	5,953	2.8	1,041	1.2	2,767	13.2	1,035	12.3	1,597	1.5
Malignant neoplasm of trachea, bronchus, and lung	200	2.0	1,714	0.8	167	0.2	976	4.7	203	2.4	568	0.5
Obstructive sleep apnea	224	2.2	3,202	1.5	1,217	1.4	710	3.4	388	4.6	1,111	1.0
*Medication use*												
LABA^a^	9,965	100.0	4,583	2.1	5,746	6.5	2,346	11.2	1,060	12.6	5,396	5.0
ICS	1,931	19.4	14,621	6.8	9,671	11.0	1,693	8.1	1,423	16.9	3,765	3.5
Fixed, combined LABA and ICS^a^	988	9.9	212,605	98.8	83,675	95.2	19,127	91.4	7,730	91.6	103,061	95.6
Short-acting beta agonist	4,399	44.1	101,481	47.2	54,461	62.0	9,430	45.0	5,348	63.3	36,641	34.0
Inhaled anticholinergics	1,811	18.2	14,311	6.7	3,422	3.9	6,900	33.0	2,510	29.7	3,290	3.1
Combined SABA and inhaled anticholinergics	935	9.4	12,679	5.9	3,983	4.5	4,560	21.8	2,040	24.2	3,031	2.8
Injected or oral corticosteroids	3,684	37.0	76,198	35.4	36,436	41.5	9,249	44.2	5,509	65.3	28,688	26.6
Leukotriene modifiers	2,241	22.5	36,924	17.2	22,577	25.7	1,914	9.1	2,283	27.0	12,391	11.5
Mast cell stabilizers	74	0.7	369	0.2	281	0.3	17	0.1	21	0.2	124	0.1
Immunoglobulin-E blockers	64	0.6	340	0.2	335	0.4	4	0.0	42	0.5	23	0.0
Xanthine derivatives	320	3.2	2,142	1.0	936	1.1	599	2.9	445	5.3	482	0.4
*Healthcare utilization*												
Ambulatory visit	9,340	93.7	204,687	95.2	87,834	100.0	20,895	99.8	8,432	99.9	96,866	89.8
Emergency department visit	2,278	22.9	46,120	21.4	20,083	22.9	6,764	32.3	3,950	46.8	17,601	16.3
Inpatient stay	1,258	12.6	18,729	8.7	6,334	7.2	5,784	27.6	3,357	39.8	4,512	4.2
Pathology/Laboratory code	6,724	67.5	140,407	65.3	58,592	66.7	15,357	73.4	6,504	77.0	66,678	61.8
Spirometry procedure	3,039	30.5	50,465	23.5	28,720	32.7	7,635	36.5	3,912	46.3	13,237	12.3
Home oxygen use procedure	783	7.9	6,704	3.1	1,461	1.7	3,402	16.3	1,214	14.4	1,410	1.3
Cardiovascular procedure	2,936	29.5	52,404	24.4	19,008	21.6	10,399	49.7	4,599	54.5	21,334	19.8
Surgical procedure	5,283	53.0	106,468	49.5	43,104	49.1	13,216	63.1	5,334	63.2	50,097	46.5
Anesthesia procedure	1,034	10.4	17,936	8.3	6,887	7.8	2,989	14.3	1,258	14.9	7,836	7.3
Visit to allergist/immunologist	1,111	11.1	17,759	8.3	14,522	16.5	311	1.5	689	8.2	3,348	3.1
Visit to pulmonologist	2,622	26.3	27,141	12.6	11,604	13.2	7,856	37.5	4,133	49.0	6,170	5.7

LABA, long-acting beta agonist; ICS, inhaled corticosteroid; COPD, chronic obstructive pulmonary disease.

^a^Including the cohort-defining dispensing.

Patients on LABAs without ICS had a higher observed prevalence of use of other pulmonary medications, including inhaled anticholinergics, leukotriene modifiers, and xanthine derivatives. Use of inhaled anticholinergics was more prevalent among patients with baseline claims for COPD, while use of leukotriene modifiers was more common among patients with claims for asthma.

We sought 640 medical records related to 520 claims consistent with asthma, COPD, both, or neither. One hundred twenty of these records were requested from alternate providers, when such an alternate was available and the initial provider was unable or unwilling to complete the request. We received medical records relating to 370 (71%) of potential cases. The Supplementary Material contains information on the reasons for non-procurement of medical records, which were generally administrative in nature. Patients whose medical records we received were slightly older than the underlying LABA cohort, but had a similar sex distribution (Table 2).

**Table 2:** Baseline age and gender of LABA initiators whose medical record was abstracted for confirmation of asthma or COPD, January 1, 2005 – December 31, 2008

	**LABA**		**LABA & ICS**		**Asthma**		**COPD**		**Asthma & COPD**		**Neither Asthma nor COPD**	
	**(N = 14)**		**(N = 356)**		**(N = 91)**		**(N = 92)**		**(N = 104)**		**(N = 83)**	
	**N**	**%**	**N**	**%**	**N**	**%**	**N**	**%**	**N**	**%**	**N**	**%**
Age												
20-39	2	14.3	49	13.8	24	26.4	4	4.4	6	5.8	17	20.5
40-64	11	78.6	242	68.0	62	68.1	58	63.0	76	73.1	57	68.7
>64	1	7.1	65	18.3	5	5.5	30	32.6	22	21.2	9	10.8
Women	8	57.1	210	59.0	57	62.6	50	54.4	60	57.7	51	61.5

LABA, long-acting beta agonist; ICS, inhaled corticosteroid; COPD, chronic obstructive pulmonary disease.

The PPV of having at least one claim for asthma (and no COPD claim) in the 6 months prior to the index date was 73.6% (CI 63.3–82.3) for confirmed asthma and 81.5% for COPD with no asthma claim (CI 72.1–88.9; Table 3). We observed that 21.2% (CI 13.8–30.3) of patients with claims for asthma and COPD were confirmed to have both conditions, while 28.9% of patients with no claims for asthma or COPD (CI 19.5–39.9) were confirmed to have asthma. Regarding the negative predictive value of claims for asthma and COPD, 61.5% (CI 50.1–71.9) of patients without these diagnoses had no evidence of the conditions in the medical record, while 28.9% (CI 19.5–39.9) had confirmed asthma and 9.6% (CI 4.1–18.1) had confirmed COPD.

**Table 3:** Confirmed cases of asthma, chronic obstructive pulmonary disease, both, or neither among the corresponding claims categories

		***Confirmed cases***							
		**Asthma**		**COPD**		**Asthma and COPD**		**Unconfirmed**	
		**N**	**%**	**N**	**%**	**N**	**%**	**N**	**%**
***Claims-identified cases***	Asthma (n = 91)	67	73.6	2	2.2	0	0.0	22	24.2
	COPD (n = 92)	6	8.7	73	81.5	2	2.2	11	7.6
	Both (n = 104)	40	38.5	35	33.7	22	21.2	7	6.7
	Neither (n = 83)	24	28.9	8	9.6	0	0.0	51	61.5

COPD, Chronic Obstructive Pulmonary Disease.

The PPV of having at least one claim for asthma decreased with increasing age, but the PPV of having of at least one claim for COPD increased with age (Table 4). Stratification on the 3 covariates with the largest individual discrimination between confirmed and unconfirmed case status resulted in higher PPVs. For instance, among patients with at least one claim for COPD and at least one dispensing of an inhaled anticholinergic medication, the PPV was 94.3% (CI 80.8–99.3). Similar findings were evident for the other empirically identified discriminators.

**Table 4:** Positive predictive value of claims for asthma, COPD, Both or Neither in the 6 Months prior to initiation of a long-acting beta agonist, Overall and stratified by empirically-identified covariates

		**Number of confirmed cases**	**Medical records received**	**Positive predictive value (%)**	**95% CI (%)**
***Claim for asthma only***					
Overall		67	91	73.6	63.3, 82.3
Age, years					
20-39		19	24	79.2	57.8, 92.9
40-64		45	62	72.6	59.8, 83.1
> 64		3	5	60.0	14.7, 94.7
Sex					
Male		25	34	73.5	55.6, 87.1
Female		42	57	73.7	60.3, 84.5
***Top 3 Variables predictive of asthma confirmation***					
Outpatient visit, moderate complexity	Yes	42	51	82.4	69.1, 91.6
	No	25	40	62.5	45.8, 77.3
Beta-adrenergic drug dispensing	Yes	53	66	80.3	68.7, 89.1
	No	14	25	56.0	34.9, 75.6
West region	Yes	18	19	94.7	74.0, 99.9
	No	49	72	68.1	56.0, 78.6
***Claim for COPD only***					
Overall		75	92	81.5	72.1, 88.9
Age, years					
20-39		2	4	50.0	6.8, 93.2
40-64		44	58	75.9	62.8, 86.1
> 64		29	30	96.7	82.8, 99.9
Sex					
Male		38	42	90.5	77.4, 97.3
Female		37	50	74.0	59.7, 86.4
***Top 3 Variables predictive of COPD confirmation***					
65+ years of age	Yes	29	30	96.7	82.8, 99.9
	No	46	62	74.2	61.5, 84.5
Inhaled anticholinergic drug dispensing	Yes	33	35	94.3	80.8, 99.3
	No	42	57	73.7	60.3, 84.5
Radiologic examination, chest, 2 views, frontal and lateral	Yes	48	54	88.9	77.4, 95.8
	No	27	38	71.1	54.1, 84.6
***Claim for Asthma and COPD (PPV of asthma only)***					
Overall		40	104	38.5	29.1, 48.5
Age, years					
20-39		5	6	83.3	35.9, 99.6
40-64		30	76	39.5	28.4, 52.4
> 64		5	22	22.7	7.8, 45.4
Sex					
Male		19	44	43.2	28.3, 59.0
Female		21	60	35.0	23.1, 48.4
***Top 3 Variables predictive of asthma only among patients with claims-based COPD and asthma***					
Chronic airway obstruction, not elsewhere classified (ICD-9 496.xx)	Yes	20	76	26.3	16.9, 37.7
	No	20	28	71.4	51.3, 86.8
General Bronchodilator Agents	Yes	6	35	17.1	6.6, 33.6
	No	34	69	49.3	37.0, 61.6
Inhaled corticosteroid dispensing	Yes	9	39	23.1	11.1, 39.3
	No	31	65	47.7	35.1, 60.5
***No claims for asthma or COPD (PPV of asthma only)***					
Overall		24	83	28.9	19.5, 39.9
Age, years					
20-39		7	17	41.2	18.4, 67.1
40-64		14	57	24.6	14.1, 37.8
> 64		3	9	33.3	7.5, 70.1
Sex					
Male		8	32	25.0	11.5, 43.4
Female		16	51	31.4	19.1, 45.9
***Top 3 Variables predictive of asthma only among patients with neither claims-based COPD nor asthma***					
Lipid panel	Yes	1	23	4.4	0.1, 21.9
	No	23	60	38.3	26.1, 51.8
Collection of venous blood by venipuncture	Yes	4	31	12.9	3.6, 29.8
	No	20	52	38.5	25.3, 53.0
Macrolides	Yes	4	29	13.8	3.9, 31.7
	No	20	54	37.0	24.3, 51.3

COPD, chronic obstructive pulmonary disease.

Stratification on clinically defined covariates resulted in some categories where the PPV was higher than the average PPV for that diagnosis (Table 5). The PPV for at least one asthma claim was increased in the presence of previous asthma medication use and lower respiratory tract infections. The PPV for at least one COPD claim was increased when a pulmonologist was the prescriber of the COPD medications and when there was a diagnosis of bronchitis or bronchiolitis. The Supplementary Material contains estimated PPVs across strata defined by combinations of variables and for a long baseline period of 12 months.

**Table 5:** Positive predictive value of claims for asthma, COPD, both or neither in the 6 months prior to initiation of a long-acting beta agonist, overall and stratified by clinically-defined covariates

	**Number of confirmed cases**	**Medical records received**	**Positive predictive value (%)**	**95% CI (%)**
***Claim for asthma only***				
Medications				
Previous LABA	7	8	87.5	47.3, 99.7
Previous ICS	12	14	85.7	57.2, 98.2
Inhaled anticholinergics	0	0	0.0	N/A
Systemic corticosteroids	31	40	77.5	61.5, 89.2
Leukotriene modifiers	24	30	80.0	61.4, 92.3
Xanthine inhibitors	3	3	100.0	29.2, 100.0
Prescriber Specialty				
Family/general provider	36	48	75.0	60.4, 86.4
Allergy/immunology	15	20	75.0	51.0, 91.3
Internal medicine	30	42	71.4	55.4, 84.3
Pulmonology	13	17	76.5	50.1, 93.2
Diagnoses				
Upper respiratory tract infections	40	49	81.6	68.0, 91.2
Lower respiratory tract infection	67	91	73.6	63.3, 82.3
Bronchitis/Bronciolitis	19	24	79.2	57.8, 93.0
Spirometry procedure	26	33	78.8	61.1, 91.0
***Claim for COPD Only***				
Medications				
Previous LABA	3	5	60.0	14.7, 94.7
Previous ICS	4	4	100.0	39.8, 100.0
Inhaled anticholinergics	33	35	94.3	80.8, 99.3
Systemic corticosteroids	29	36	80.6	64.0, 91.8
Leukotriene modifiers	5	9	55.6	21.2, 86.3
Xanthine inhibitors	2	2	100.0	16.8, 100.0
Prescriber Specialty				
Family/general provider	40	48	83.3	69.8, 92.5
Allergy/immunology	0	3	0.0	0.0, 70.6
Internal medicine	34	42	81.0	65.9, 91.4
Pulmonology	29	32	90.6	75.0, 98.0
Diagnoses				
Upper respiratory tract infections	12	20	60.0	36.1, 80.9
Lower respiratory tract infection	75	92	81.5	72.1, 88.9
Bronchitis/Bronciolitis	51	59	86.4	75.0, 94.0
Spirometry procedure	32	39	82.1	66.5, 92.5
***Claim for asthma and COPD (PPV of asthma only)***				
Medications				
Previous LABA	3	12	25.0	5.5, 57.2
Previous ICS	4	14	28.6	8.4, 58.1
Inhaled anticholinergics	9	39	23.1	11.1, 39.3
Systemic corticosteroids	27	70	38.6	27.1, 51.0
Leukotriene modifiers	10	30	33.3	17.3, 52.8
Xanthine inhibitors	2	6	33.3	4.3, 77.7
Prescriber Specialty				
Family/general provider	22	67	32.8	21.8, 45.4
Allergy/immunology	7	10	70.0	34.8, 93.3
Internal medicine	25	59	42.4	29.6, 55.9
Pulmonology	19	52	36.5	23.6, 51.0
Diagnoses				
Upper respiratory tract infections	20	43	46.5	31.2, 62.3
Lower respiratory tract infection	40	104	38.5	29.1, 48.5
Bronchitis/Bronciolitis	28	72	38.9	27.6, 51.1
Spirometry procedure	21	53	39.6	26.5, 54.0
***No claims for asthma or COPD (PPV of asthma only)***				
Medications				
Previous LABA	0	4	0.0	0.0, 60.2
Previous ICS	0	2	0.0	0.0, 84.2
Inhaled anticholinergics	0	2	0.0	0.0, 84.2
Systemic corticosteroids	9	28	32.1	15.9, 52.4
Leukotriene modifiers	5	11	45.5	16.7, 76.6
Xanthine inhibitors	0	0	0.0	N/A
Prescriber specialty				
Family/general provider	12	48	25.0	13.6, 39.6
Allergy/immunology	1	1	100.0	2.5, 100.0
Internal medicine	7	28	25.0	10.7, 44.9
Pulmonology	3	7	42.9	9.9, 81.6
Diagnoses				
Upper respiratory tract infections	5	30	16.7	5.6, 34.7
Lower respiratory tract infection	6	33	18.2	7.0, 35.5
Bronchitis/Bronchiolitis	5	29	17.2	5.8, 35.8
Spirometry procedure	3	8	37.5	8.5, 75.5

The multinomial logistic regression modeling identified several variables that improved prediction of true case status through interaction with the claims definitions. The variables “symptoms involving respiratory system and other chest symptoms” (ICD-9 786.xx), the number of baseline drug dispensings, and age > 40 years improved prediction of COPD only. The number of drug dispensings also improved prediction of asthma only and both asthma and COPD relative to neither. The presence of a baseline dispensing of a beta-adrenergic drug and the diagnosis “other forms of chronic ischemic heart disease” (ICD-9 414.xx) improved prediction of true asthma only. No other variables arose as significant predictors.

## Discussion

Subsets of LABA initiators with asthma, COPD, and both conditions can be identified and differentiated using claims data, although categorization of the remaining patients is largely infeasible. Within strata of selected covariates, the claims showed better predictive ability to identify asthma only among patients with claims for asthma or claims for both asthma and COPD, and to identify COPD among patients with claims for COPD. Among patients without claims for asthma or COPD, there was no subset within which the PPV exceeded 50%, meaning the classification of patients who do not have a claim indicating asthma or COPD remains uncertain. Additionally, requiring the presence of claims for asthma or COPD resulted in a population in which nearly 25% of persons appear to not have the condition of interest.

That a substantial fraction of the LABA users did not have a claim associated with asthma or COPD is problematic in that it leaves a large fraction of patients unclassified in the data. Similarly, multiple diagnoses of asthma or COPD were rare, limiting our ability to refine the algorithms by requiring more than one diagnosis for the condition of interest. Both of these findings are consistent with previous experience with health plan data. Loughlin and colleagues found with tegaserod, a drug with a clear indication (irritable bowel syndrome), only 32% of tegaserod initiators had a claim for irritable bowel syndrome in the 6-month period preceding tegaserod initiation [12]. To address this expected feature of the data, we abstracted medical records on a subset of LABA initiators with no claims for asthma or COPD, and confirmed their actual case status. The resulting data provide an estimate of the distribution of actual asthma and COPD among those without a claims-diagnosis. Nevertheless, a large fraction of patients remain unclassified. In studies of off-label use, this limitation means that there will be overestimation of off-label use (under the assumption that some of the unclassified patients have the “on-label” indication). In studies where there is a desire to exclude patients with asthma or COPD, it may be infeasible to completely exclude these individuals.

Our findings were qualitatively similar to those in a study of the predictive ability of the Lovelace Health Plan data, a staff- and network-model health maintenance organization, for identifying preclinical COPD [8]. In that study, a small number of claims-based variables predicted the presence of preclinical COPD with a PPV of 23% to 39% depending on the definition used. The higher PPVs observed in our study probably reflect our focus on diagnosed COPD, while the Lovelace study screened for preclinical disease.

We did not directly assess the sensitivity of the claims definition; however, the small number of LABA users observed to have baseline claims for asthma or COPD overall suggest that the sensitivity of the diagnosis codes on claims may be low since nearly all LABA users would be expected to have one of these conditions. Other limitations of this study include the use of medical records as a gold standard measure of asthma and COPD. This approach requires the assumption that the information needed for asthma and COPD classification is present and not differentially present across the strata of the predictive variables. If the incorrect diagnosis is listed in the medical record or the medical record is incomplete, then the present estimates of the PPV could be biased. The bias would likely result in an underestimate of the PPV if the medical records are incomplete, since we required that cases have the diagnostic criteria affirmatively listed in their record.

These data may not be generalizable to populations of patients who do not use LABAs and caution is warranted for applications of these data to patients with different treatment patterns. Since our objective was to study LABA initiators, we did not collected data on non-users of LABA. Similarly, these data may not generalize to populations other than the commercially insured, but stratifying this population according to relevant characteristics and providing the PPV within strata, improves the external application of these data for case identification [13]. Although these characteristics for stratification are based on health insurance claims in this study were, and so are subject to misclassification on the clinical constructs of interest, this type of misclassification is frequently thought to be non-differential with respect to other claims-based variables so that the most plausible direction of misclassification bias would be toward showing no difference in the PPV across these variables [14]. In some strata of predictive covariates, the data were sparse, which resulted in uncertainty around some PPV estimates. Additionally, we evaluated the performance of our own measure of asthma and COPD. Therefore, these data may provide limited information on the validity of other disease definitions.

A major strength of this study is the large source population, and its reflection of routine clinical practice across the US. These data provide adequate statistical power for the study across a number of stratification variables, and improves its generalizability.

Because of low sensitivity, the claims data do not seem sufficient to definitively identify asthma alone without sacrificing the PPV. However, this study provides estimates of the fraction of patients with confirmed asthma that one might expect from studies identified on the basis of claims alone. For subsets of patients with claims for asthma or COPD, the claims have fairly high predictive ability for identifying actual diagnoses. Therefore, a reasonable use of these data for LABA risk management is to estimate the fraction of patients with asthma through application of the observed distributions from this study, and to identify subsets of these patients based on the presence of at least one additional predictive covariate to achieve a higher degree of specificity. This use of the data would allow for periodic tracking of the fraction of potential off-label use among a subset of patients likely to have asthma only, while at the same time applying the observed (confirmed) distribution of asthma only from this study for broader context.

## Conclusions

In summary, the results from this study suggest that it is feasible to differentiate subsets of LABA initiators into categories that might represent on- vs. off-label use of LABAs. It is not possible to categorize all LABA initiators with respect to these diagnoses. Safety surveillance for off-label use of LABAs must account for this limitation. Nevertheless, these data provide confirmed distributions of actual diagnoses that can be extrapolated to future surveillance studies and facilitate improved identification of subsets of patients with asthma, COPD, both, or neither on the basis of health insurance claims data. Implementation of simple algorithms that combine the presence of a diagnosis code for asthma or COPD with the presence or absence of a number of single PPV-predictive covariates would allow for periodic tracking of off-label LABA prescribing among subsets of all LABA users. The specific algorithm chosen should be targeted to the application at hand.

## Abbreviations

LABA: Long-acting β-agonist; COPD: Chronic obstructive pulmonary disease; ICS: Inhaled corticosteroids; REMS: Risk evaluation and mitigation strategies; ICD-9: International Classification of Disease, 9th Revision; CPT: Common procedural terminology; HCPCS: Centers for Medicare and Medicaid Services Common Procedure Coding System; NDC: National drug code; GOLD: Global Initiative for Chronic Obstructive Lung Disease; PPV: Positive predictive value; CI: 95% confidence interval; PHI: Protected health information.

## Competing interest

Sponsor: This study was funded by a research contract between Optum Epidemiology and Novartis Pharmaceuticals. The contract granted Optum oversight of the study conduct, reporting, and interpretation, as well as final wording of any resulting manuscripts. Novartis is the maker of formoterol, a product included in this study.

Conflict of Interest Statement: Authors Dore, Ziyadeh, Clifford, Norman and Seeger were employees of Optum, a division of UnitedHealth Group at the time this research was conducted. Dr. Cai was an employee of Novartis Pharmaceuticals at the time this research was conducted.

Prior Postings: This work was presented in poster form at the 2011 International Conference on Pharmacoepidemiology and Therapeutic Risk Management, Chicago, IL, USA.

## Authors’ contributions

DD supervised the study analysis and wrote the manuscript. NZ wrote the protocol helped to draft the manuscript and interpret the results. BC conceived of the study and participated in interpretation of data and revision of the manuscript. CRC and HN performed the statistical analysis and participated in the interpretation of data. JS conceived of the study and participated in interpretation of data and revision of the manuscript. All authors read and approved the final manuscript.

## Pre-publication history

The pre-publication history for this paper can be accessed here:

http://www.biomedcentral.com/1471-2466/14/47/prepub

## Supplementary Material

Additional file 1 Identification of Prevalent Asthma and Chronic Obstructive Pulmonary Disease among Initiators of Long-Acting β-Agonists in Health Insurance Claims Data. Click here for file
