# Supplementary material for: A cross-sectional study of the identification of prevalent asthma and chronic obstructive pulmonary disease among initiators of long-acting β-agonists in health insurance claims data
Source: BMC Pulm Med. 2014 Mar 19;14:47. doi: 10.1186/1471-2466-14-47 (PMC4000130; doi:10.1186/1471-2466-14-47)

Supplementary Material

Identification of Prevalent Asthma and Chronic Obstructive Pulmonary Disease among Initiators of Long-Acting β-Agonists in Health Insurance Claims Data

David D. Dore, PharmD, PhD[1,2,3,4]; Najat Ziyadeh, MA, MPH[4]; Bin Cai, MD, MS, PhD[5]; C. Robin Clifford, MS[4]; Heather Norman, MS[4]; John D. Seeger, PharmD, DrPH[4,6]

[1]*Department of Health Services, Policy & Practice; Brown School of Public Health, Providence, RI*
[2]*Department of Epidemiology; Brown School of Public Health, Providence, RI*
[3]*Center for Gerontology and Health Care Research, Brown School of Public Health, Providence, RI*
[4]*Optum Epidemiology, Waltham, MA*
[5]*Novartis Pharmaceuticals, East Hanover, NJ*
[6]*Division of Pharmacoepidemiology and Pharmacoeconomics, Brigham and Women's Hospital/Harvard Medical School, Boston, MA*

Corresponding Author: David D. Dore, PharmD, PhD
Brown University, Box G-121-7
121 South Main Street
Providence, RI 02903
Tel: 401-863-1980
Email: david_dore@brown.edu

In this supplement, Figure 1 lists the reasons for non-procurement of medical records. We received medical records relating to 370 (71%) of potential cases and non-procurement of medical records occurred mostly because of administrative barriers. Table 1 includes information on severity classifications of confirmed asthma and COPD. The classification procedures are outlined in Figure 2. An external physician, blinded to study medications, adjudicated the presence of asthma and COPD, and classified the diagnosis into categories of severity using the Global Initiative for Chronic Obstructive Lung Disease (GOLD) criteria for COPD[1] and the third Expert Panel Report (EPR) of Guidelines on Asthma[2] for asthma. The COPD designations included[1]:

I: Mild COPD	• $FEV_1/FVC < 0.7$<br>• $FEV_1 \geq 80\%$ predicted	At this stage, the patient may not be aware that their lung function is abnormal.
II: Moderate COPD	• $FEV_1/FVC < 0.7$<br>• $50\% \leq FEV_1 < 80\%$ predicted	Symptoms usually progress at this stage, with shortness of breath typically developing on exertion.
III: Severe COPD	• $FEV_1/FVC < 0.7$<br>• $30\% \leq FEV_1 < 50\%$	Shortness of breath predicted typically worsens at this stage and often limits patients' daily activities. Exacerbations are especially seen beginning at this stage.
IV: Very Severe COPD	• $FEV_1/FVC < 0.7$<br>• $FEV_1 < 30\%$ predicted *or* $FEV_1 < 50\%$ predicted plus chronic respiratory failure	At this stage, quality of life is very appreciably impaired and exacerbations may be life-threatening.

Classification of asthma by severity was based on frequency and severity of asthma symptoms, along with peak flow readings. Levels are referred to as steps, as follows:

Step 1: Mild Intermittent. At this level, asthma symptoms occur less than 2 times a week during waking hours and less than twice a month during the night. In between asthma attacks, no symptoms occur at all, and the attacks themselves are generally brief, though their intensity can vary. Peak flow variability is less than 20 percent.

Step 2: Mild Persistent. At this level, asthma symptoms are occurring more than twice a week, but not as often as daily. They may occasionally wake the patient up at night, but that happens

---

[1] Global Initiative for Chronic Obstructive Lung Disease. Global strategy for the diagnosis, management, and prevention of chronic obstructive pulmonary disease. (Updated 2007). www.goldcopd.org. Accessed on 14 July 2009.

[2] National Heart, Lung, and Blood Institute, 2007. National Asthma Education and Prevention Program, Expert Panel Report 3: Guidelines for the Diagnosis and Management of Asthma. Full Report 2007. www.nhlbi.nih.gov/guidelines/asthma/asthgdln.pdf. Accessed 15 July 2009.

less than 2 times a month. Asthma attacks may interfere with activity temporarily. Peak flow tends to be more than 20—but less than 30—percent.

Step 3: Moderate Persistent. At this level, asthma is starting to interfere more with daily living. Symptoms are cropping up every single day, and the patient may need to use a quick-relief inhaler daily. Asthma attacks are occurring at least twice a week and often interfere with activity. They may last for days at a time. The patient is probably also waking up one or more times a week with symptoms. Peak flow rate varies by more than 30 percent.

Step 4: Severe Persistent. This is the most severe form of asthma and at this level, symptoms are basically continuous. Activity is severely limited and asthma attacks and night symptoms are frequent. Peak flow varies by more than 30 percent.

The severity of asthma and COPD among patients who were confirmed to have both diagnoses was classified for each condition. The medical record abstraction covered a period of 3 months before and after the index claim for asthma and COPD. The adjudication was based on a series of yes-no questions; reviewers were not required to list supporting reasons for adjudication decisions. Confirmation required the affirmative listing of the case criteria in the medical records.

Adjudication Questions

Question 1: Was there a recorded diagnosis of COPD by the treating physician?

Question 2: Is this patient's COPD mild?

Question 3: Is this patient's COPD moderate?

Question 4: Is this patient's COPD severe?

Question 5: Is this patient's COPD very severe?

Question 6: Was there a recorded diagnosis of asthma by the treating physician?

Question 7: Is this patient's asthma intermittent?

Question 8: Is this patient's asthma mild persistent?

Question 9: Is this patient's asthma moderate persistent?

Question 10: Is this patient's asthma severe persistent?

Table 2 of this supplement is a tabulation of pre-determined and empirically identified covariates chosen with the aim of identifying variables that discriminate between confirmed and unconfirmed case status among patients with claims for asthma or COPD. The table also shows the distribution of covariate patterns defined by 3 covariates with the largest absolute difference in prevalence across patients with confirmed and unconfirmed asthma among patients with claims-based asthma only, both asthma and COPD, or neither asthma nor COPD. For patients with claims-based COPD only, we were interested in the fraction with true COPD and we compared categories of confirmed COPD vs. unconfirmed COPD to identify the 3 most predictive covariates. Note that these "top" empirical variables are numbered in the table when listed singly and referred to by their numbers when listed as part of a covariate pattern.

Table 3 of this supplement shows the positive predictive value of claims-identification of asthma, COPD, both, or neither stratified by the variables in Table 2 and using a six month look-back period. Table 4 contains the same data for a twelve month look-back period. Some of this information is repeated from Tables 4 and 5 in the main publication. Covariate patters are similarly referred to by number-labels.

Table 1: Asthma, COPD, Both or Neither Occurring in Baseline Confirmed by Adjudication Among LABA (N=9,965) and LABA & ICS (N=215,114) Initiators by Severity of Disease

Initiation Period: 01 January 2005 - 31 December 2008

**Medical Records Received**	**LABA (N=14)**		**LABA & ICS (N=356)**	
	N	%	N	%
Among Patients with Claims-Based Asthma Only	2	14.29	89	25.00
**Confirmed Asthma[1]**	1	7.14	66	18.54
I: Mild Intermittent	0	0.00	22	6.18
II: Mild Persistent	0	0.00	12	3.37
III: Moderate Persistent	0	0.00	22	6.18
IV: Severe Persistent	1	7.14	10	2.81
**Confirmed COPD[2]**	0	0.00	2	0.56
**Confirmed Asthma & COPD[1, 2]**	0	0.00	0	0.00
**Confirmed neither Asthma nor COPD[1, 2]**	1	7.14	21	5.90
Among Patients with Claims-Based COPD Only	4	28.57	88	24.72
**Confirmed COPD[2]**	2	14.29	73	20.51
I: Mild	0	0.00	21	5.90
II: Moderate	1	7.14	25	7.02
III: Severe	1	7.14	22	6.18
IV: Very Severe	0	0.00	5	1.40
**Confirmed Asthma[1]**	1	7.14	7	1.97
**Confirmed Asthma & COPD[1, 2]**	0	0.00	2	0.56
**Confirmed neither Asthma nor COPD[1, 2]**	1	7.14	10	2.81
Among Patients with Claims-Based Asthma & COPD[3]	4	28.57	100	28.09
**Confirmed Asthma & COPD[1, 2]**	0	0.00	22	6.18
Asthma (among patients with asthma and COPD)	0	0.00	22	6.18
I: Mild Intermittent	0	0.00	6	1.69
II: Mild Persistent	0	0.00	1	0.28
III: Moderate Persistent	0	0.00	6	1.69
IV: Severe Persistent	0	0.00	9	2.53
COPD (among patients with asthma and COPD)	0	0.00	22	6.18
I: Mild	0	0.00	6	1.69
II: Moderate	0	0.00	8	2.25
III: Severe	0	0.00	7	1.97
IV: Very Severe	0	0.00	1	0.28
**Confirmed Asthma Only[1]**	1	7.14	39	10.96
**Confirmed COPD Only[2]**	2	14.29	33	9.27
All Asthma (with or without comorbid COPD)	1	7.14	61	17.13
I: Mild Intermittent	1	7.14	20	5.62
II: Mild Persistent	0	0.00	4	1.12
III: Moderate Persistent	0	0.00	15	4.21
IV: Severe Persistent	0	0.00	22	6.18
All COPD (with or without comorbid asthma)	2	14.29	55	15.45
I: Mild	0	0.00	15	4.21
II: Moderate	0	0.00	15	4.21
III: Severe	1	7.14	22	6.18
IV: Very Severe	1	7.14	3	0.84
**Confirmed neither Asthma nor COPD[1, 2]**	1	7.14	6	1.69
Among Patients with Claims-Based neither Asthma nor COPD	4	28.57	79	22.20
**Confirmed neither Asthma nor COPD[1, 2]**	4	28.57	47	13.20
**Confirmed Asthma[1]**	0	0.00	24	6.74

Table 1: Asthma, COPD, Both or Neither Occurring in Baseline Confirmed by Adjudication Among LABA (N=9,965) and LABA & ICS (N=215,114) Initiators by Severity of Disease

Initiation Period: 01 January 2005 - 31 December 2008

**Medical Records Received**	**LABA (N=14)**		**LABA & ICS (N=356)**	
	N	%	N	%
I: Mild Intermittent	0	0.00	15	4.21
II: Mild Persistent	0	0.00	6	1.69
III: Moderate Persistent	0	0.00	2	0.56
IV: Severe Persistent	0	0.00	1	0.28
**Confirmed COPD[2]**	0	0.00	8	2.25
I: Mild	0	0.00	2	0.56
II: Moderate	0	0.00	1	0.28
III: Severe	0	0.00	5	1.40
IV: Very Severe	0	0.00	0	0.00
**Confirmed Asthma & COPD[1, 2]**	0	0.00	0	0.00

Abbreviations: LABA, long-acting beta$_2$ agonist; ICS, inhaled corticosteroid; COPD, chronic obstructive pulmonary disease

[1]Expert Panel Report (EPR) 3 Guidelines on Asthma, developed by an expert panel commissioned by the National Asthma Education and Prevention Program (NAEPP) Coordinating Committee (CC), coordinated by the National Heart, Lung, and Blood Institute (NHLBI) of the National Institutes of Health (NIH)

[2]The Global Initiative for Chronic Obstructive Lung Disease (GOLD) criteria

[3]Excludes patients with confirmed asthma or COPD (6 patients)

Table 2: Characterization of Adjudicated Cases of Asthma, COPD, or Both with respect to Claims for Medical Services in the 6 Months Prior to Cohort Entry that may Predict Potential Cases

Initiation Period: 01 January 2005 - 31 December 2008

	Claims-based Asthma				Claims-based COPD				Claims-based COPD & Asthma				Claims-based Neither Asthma nor COPD			
	Confirmed Asthma		Unconfirmed Asthma		Confirmed COPD		Unconfirmed COPD		Confirmed Asthma Only		Unconfirmed Asthma Only		Confirmed Asthma Only		Unconfirmed Asthma Only	
	N=67		N=24		N=75		N=17		N=40		N=64		N=24		N=59	
	n	%	n	%	n	%	n	%	n	%	n	%	n	%	n	%
20-39 years old	19	28.36	5	20.83	2	2.67	2	11.76	5	12.50	1	1.56	7	29.17	10	16.95
40-64 years old	45	67.16	17	70.83	44	58.67	14	82.35	30	75.00	46	71.88	14	58.33	43	72.88
> 64 years old	3	4.48	2	8.33	29	38.67	1	5.88	5	12.50	17	26.56	3	12.50	6	10.17
Male	25	37.31	9	37.50	38	50.67	4	23.53	19	47.50	25	39.06	8	33.33	24	40.68
Female	42	62.69	15	62.50	37	49.33	13	76.47	21	52.50	39	60.94	16	66.67	35	59.32
Top 5 Variables Predictive of Asthma Confirmation																
1. Outpatient visit, moderate complexity (CPT 99214)	42	62.69	9	37.50	46	61.33	14	82.35	30	75.00	53	82.81	13	54.17	33	55.93
2. Beta-adrenergic drug dispensing	53	79.10	13	54.17	45	60.00	10	58.82	31	77.50	49	76.56	9	37.50	23	38.98
3. West region	18	26.87	1	4.17	11	14.67	1	5.88	6	15.00	7	10.94	5	20.83	10	16.95
4. Short-acting beta agonist dispensing	51	76.12	13	54.17	34	45.33	8	47.06	25	62.50	41	64.06	9	37.50	20	33.90
5. General medical examination (ICD-9 V70)	14	20.90	0	0.00	11	14.67	0	0.00	2	5.00	10	15.63	1	4.17	7	11.86
Top 5 Variables Predictive of COPD Confirmation																
6. 65+ years of age	3	4.48	2	8.33	29	38.67	1	5.88	5	12.50	17	26.56	3	12.50	6	10.17
7. Inhaled anticholinergic drug dispensing	0	0.00	0	0.00	33	44.00	2	11.76	9	22.50	30	46.88	0	0.00	2	3.39
8. Radiologic examination, chest, 2 views, frontal and lateral (CPT 71020)	22	32.84	10	41.67	48	64.00	6	35.29	25	62.50	45	70.31	6	25.00	20	33.90
9. Rhinitis (ICD-9 477.xx)	24	35.82	8	33.33	5	6.67	6	35.29	14	35.00	15	23.44	2	8.33	13	22.03
10. Gender Male	25	37.31	9	37.50	38	50.67	4	23.53	19	47.50	25	39.06	8	33.33	24	40.68
Top 5 Variables Predictive of Asthma Only Among Patients with Claims-based COPD and Asthma																
11. Chronic airway obstruction, not elsewhere classified (ICD-9 496.xx)	0	0.00	0	0.00	59	78.67	10	58.82	20	50.00	56	87.50	0	0.00	0	0.00
12. General Bronchodilator Agents	0	0.00	0	0.00	33	44.00	2	11.76	6	15.00	29	45.31	0	0.00	0	0.00
13. Inhaled Anticholinergic drug dispensing	0	0.00	0	0.00	33	44.00	2	11.76	9	22.50	30	46.88	0	0.00	2	3.39
14. Essential hypertension (ICD-9 401.xx)	18	26.87	7	29.17	35	46.67	5	29.41	16	40.00	40	62.50	6	25.00	17	28.81
15. Drugs Administered Other than Oral Method	11	16.42	4	16.67	25	33.33	4	23.53	13	32.50	34	53.13	6	25.00	19	32.20
Top 5 Variables Predictive of Asthma Only Among Patients with Neither Claims-based COPD nor Asthma																
16. Lipid panel (CPT 80061)	18	26.87	4	16.67	26	34.67	9	52.94	11	27.50	18	28.13	1	4.17	22	37.29
17. Collection of venous blood by venipuncture (CPT 36415)	24	35.82	6	25.00	31	41.33	9	52.94	13	32.50	22	34.38	4	16.67	27	45.76
18. Macrolides	24	35.82	7	29.17	19	25.33	4	23.53	22	55.00	23	35.94	4	16.67	25	42.37
19. Nonoperative Therapeutic Cardiovascular Procedure	24	35.82	6	25.00	31	41.33	9	52.94	13	32.50	22	34.38	5	20.83	27	45.76

Table 2: Characterization of Adjudicated Cases of Asthma, COPD, or Both with respect to Claims for Medical Services in the 6 Months Prior to Cohort Entry that may Predict Potential Cases

Initiation Period: 01 January 2005 - 31 December 2008

	Claims-based Asthma				Claims-based COPD				Claims-based COPD & Asthma				Claims-based Neither Asthma nor COPD			
	Confirmed Asthma		Unconfirmed Asthma		Confirmed COPD		Unconfirmed COPD		Confirmed Asthma Only		Unconfirmed Asthma Only		Confirmed Asthma Only		Unconfirmed Asthma Only	
	N=67		N=24		N=75		N=17		N=40		N=64		N=24		N=59	
	n	%	n	%	n	%	n	%	n	%	n	%	n	%	n	%
20. Special investigations and examinations (ICD-9 V72)	10	14.93	5	20.83	17	22.67	4	23.53	7	17.50	15	23.44	1	4.17	17	28.81
Covariate Patterns, Top 3 Asthma Variables																
Variable 1, Variable 2, Variable 3[1]																
No, No, No	7	10.45	8	33.33	12	16.00	2	11.76	1	2.50	3	4.69	5	20.83	17	28.81
No, No, Yes	0	0.00	0	0.00	2	2.67	0	0.00					1	4.17	3	5.08
No, Yes, No	11	16.42	6	25.00	14	18.67	1	5.88	8	20.00	8	12.50	5	20.83	5	8.47
No, Yes, Yes	7	10.45	1	4.17	1	1.33	0	0.00	1	2.50	0	0.00	0	0.00	1	1.69
Yes, No, No	4	5.97	3	12.50	13	17.33	5	29.41	5	12.50	11	17.19	6	25.00	13	22.03
Yes, No, Yes	3	4.48	0	0.00	3	4.00	0	0.00	3	7.50	1	1.56	3	12.50	3	5.08
Yes, Yes, No	27	40.30	6	25.00	25	33.33	8	47.06	20	50.00	35	54.69	3	12.50	14	23.73
Yes, Yes, Yes	8	11.94	0	0.00	5	6.67	1	5.88	2	5.00	6	9.38	1	4.17	3	5.08
Covariate Patterns, Top 3 COPD Variables																
Variable 6, Variable 7, Variable 8[1]																
No, No, No	42	62.69	13	54.17	10	13.33	9	52.94	12	30.00	4	6.25	15	62.50	32	54.24
No, No, Yes	22	32.84	9	37.50	16	21.33	5	29.41	16	40.00	19	29.69	6	25.00	19	32.20
No, Yes, No	0	0.00	0	0.00	6	8.00	1	5.88	2	5.00	9	14.06	0	0.00	2	3.39
No, Yes, Yes	0	0.00	0	0.00	14	18.67	1	5.88	5	12.50	15	23.44	0	0.00	0	0.00
Yes, No, No	3	4.48	1	4.17	7	9.33	1	5.88	1	2.50	3	4.69	3	12.50	5	8.47
Yes, No, Yes	0	0.00	1	4.17	9	12.00	0	0.00	2	5.00	8	12.50	0	0.00	1	1.69
Yes, Yes, No	0	0.00	0	0.00	4	5.33	0	0.00	0	0.00	3	4.69	0	0.00	0	0.00
Yes, Yes, Yes	0	0.00	0	0.00	9	12.00	0	0.00	2	5.00	3	4.69	0	0.00	0	0.00
Covariate Patterns, Top 3 Asthma and COPD Variables																
Variable 11, Variable 12, Variable 13[1]																
No, No, No	67	100.00	24	100.00	10	13.33	7	41.18	17	42.50	6	9.38	24	100.00	57	96.61
No, No, Yes	0	0.00	0	0.00	0	0.00	0	0.00	2	5.00	0	0.00	0	0.00	2	3.39
No, Yes, No	0	0.00	0	0.00	0	0.00	0	0.00	0	0.00	0	0.00	0	0.00	0	0.00
No, Yes, Yes	0	0.00	0	0.00	6	8.00	0	0.00	1	2.50	2	3.13	0	0.00	0	0.00
Yes, No, No	0	0.00	0	0.00	32	42.67	8	47.06	14	35.00	28	43.75	0	0.00	0	0.00
Yes, No, Yes	0	0.00	0	0.00	0	0.00	0	0.00	1	2.50	1	1.56	0	0.00	0	0.00
Yes, Yes, No	0	0.00	0	0.00	0	0.00	0	0.00	0	0.00	0	0.00	0	0.00	0	0.00
Yes, Yes, Yes	0	0.00	0	0.00	27	36.00	2	11.76	5	12.50	27	42.19	0	0.00	0	0.00
Covariate Patterns, Top 3 Neither Asthma nor COPD Variables																
Variable 16, Variable 17, Variable 18[1]																
No, No, No	25	37.31	13	54.17	32	42.67	5	29.41	9	22.50	24	37.50	16	66.67	17	28.81
No, No, Yes	11	16.42	4	16.67	8	10.67	2	11.76	13	32.50	11	17.19	4	16.67	11	18.64
No, Yes, No	9	13.43	1	4.17	6	8.00	1	5.88	4	10.00	7	10.94	3	12.50	6	10.17
No, Yes, Yes	4	5.97	2	8.33	3	4.00	0	0.00	3	7.50	4	6.25	0	0.00	3	5.08
Yes, No, No	3	4.48	0	0.00	3	4.00	0	0.00	2	5.00	2	3.13	0	0.00	2	3.39

Table 2: Characterization of Adjudicated Cases of Asthma, COPD, or Both with respect to Claims for Medical Services in the 6 Months Prior to Cohort Entry that may Predict Potential Cases

Initiation Period: 01 January 2005 - 31 December 2008

	Claims-based Asthma				Claims-based COPD				Claims-based COPD & Asthma				Claims-based Neither Asthma nor COPD			
	Confirmed Asthma		Unconfirmed Asthma		Confirmed COPD		Unconfirmed COPD		Confirmed Asthma Only		Unconfirmed Asthma Only		Confirmed Asthma Only		Unconfirmed Asthma Only	
	N=67		N=24		N=75		N=17		N=40		N=64		N=24		N=59	
	n	%	n	%	n	%	n	%	n	%	n	%	n	%	n	%
Yes, No, Yes	4	5.97	1	4.17	1	1.33	1	5.88	3	7.50	5	7.81	0	0.00	2	3.39
Yes, Yes, No	6	8.96	3	12.50	15	20.00	7	41.18	3	7.50	8	12.50	1	4.17	9	15.25
Yes, Yes, Yes	5	7.46	0	0.00	7	9.33	1	5.88	3	7.50	3	4.69	0	0.00	9	15.25
Pre-defined Covariates																
Medications:																
Previous LABA	7	10.45	1	4.17	3	4.00	2	11.76	3	7.50	9	14.06	0	0.00	4	6.78
Previous ICS	12	17.91	2	8.33	4	5.33	0	0.00	4	10.00	10	15.63	0	0.00	2	3.39
Inhaled anticholinergics	0	0.00	0	0.00	33	44.00	2	11.76	9	22.50	30	46.88	0	0.00	2	3.39
Systemic corticosteroids	31	46.27	9	37.50	29	38.67	7	41.18	27	67.50	43	67.19	9	37.50	19	32.20
Leukotriene modifiers	24	35.82	6	25.00	5	6.67	4	23.53	10	25.00	20	31.25	5	20.83	6	10.17
Mast cell stabilizers	1	1.49	1	4.17	0	0.00	1	5.88	0	0.00	0	0.00	0	0.00	0	0.00
IgE blockers	0	0.00	0	0.00	0	0.00	0	0.00	0	0.00	0	0.00	0	0.00	0	0.00
Xanthine inhibitors	3	4.48	0	0.00	2	2.67	0	0.00	2	5.00	4	6.25	0	0.00	0	0.00
Prescriber Specialty:																
Family/general provider	36	53.73	12	50.00	40	53.33	8	47.06	22	55.00	45	70.31	12	50.00	36	61.02
Allergy/immunology	15	22.39	5	20.83	0	0.00	3	17.65	7	17.50	3	4.69	1	4.17	0	0.00
Prescriber Specialty, cont.:																
Internal medicine	30	44.78	12	50.00	34	45.33	8	47.06	25	62.50	34	53.13	7	29.17	21	35.59
Pulmonology	13	19.40	4	16.67	29	38.67	3	17.65	19	47.50	33	51.56	3	12.50	4	6.78
Allied health professional	0	0.00	0	0.00	2	2.67	0	0.00	1	2.50	1	1.56	0	0.00	0	0.00
Other	12	17.91	7	29.17	16	21.33	4	23.53	10	25.00	11	17.19	5	20.83	20	33.90
Diagnoses:																
Upper respiratory tract infections	40	59.70	9	37.50	12	16.00	8	47.06	20	50.00	23	35.94	5	20.83	25	42.37
Lower respiratory tract infection	67	100.00	24	100.00	75	100.00	17	100.00	40	100.00	64	100.00	6	25.00	27	45.76
Bronchitis/Bronchiolitis	19	28.36	5	20.83	51	68.00	8	47.06	28	70.00	44	68.75	5	20.83	24	40.68
Congestive heart failure	1	1.49	0	0.00	13	17.33	0	0.00	3	7.50	6	9.38	0	0.00	1	1.69
Other:																
Spirometry procedure	26	38.81	7	29.17	32	42.67	7	41.18	21	52.50	32	50.00	3	12.50	5	8.47

Abbreviations: COPD, chronic obstructive pulmonary disease; ICD, International Classification of Disease; CPT, current procedural terminology

[1]Numbered above. Yes indicates presence of that Variable, No indicates absence of that Variable.

Table 3: Positive Predictive Value of Claims for Asthma, COPD, or Both in the 6 Months Prior to Cohort Entry

	Number of Confirmed Cases	Medical Records Received	Positive Predictive Value
Claim for Asthma Only			
Overall	67	91	73.6
Age, years			
20-39	19	24	79.2
40-64	45	62	72.6
> 64	3	5	60.0
Sex			
Male	25	34	73.5
Female	42	57	73.7
Top 3 Variables Predictive of Asthma Confirmation			
1. Outpatient visit, moderate complexity (CPT 99214)			
Yes	42	51	82.4
No	25	40	62.5
2. Beta-adrenergic drug dispensing			
Yes	53	66	80.3
No	14	25	56.0
3. West region			
Yes	18	19	94.7
No	49	72	68.1
Covariate Patterns, Top 3 Asthma Variables			
Variable1, Variable 2, Variable 3[1]			
No, No, No	7	15	46.7
No, No, Yes	0	0	0.0
No, Yes, No	11	17	64.7
No, Yes, Yes	7	8	87.5
Yes, No, No	4	7	57.1
Yes, No, Yes	3	3	100.0
Yes, Yes, No	27	33	81.8
Yes, Yes, Yes	8	8	100.0
Pre-defined Covariates			
Medications:			
Previous LABA			
Yes	7	8	87.5
No	60	83	72.3
Previous ICS			
Yes	12	14	85.7
No	55	77	71.4
Inhaled anticholinergics			
Yes	0	0	0.0
No	67	91	73.6
Systemic corticosteroids			
Yes	31	40	77.5
No	36	51	70.6
Leukotriene modifiers			
Yes	24	30	80.0
No	43	61	70.5
Mast cell stabilizers			
Yes	1	2	50.0
No	66	89	74.2
Medications, cont.:			
IgE blockers			
Yes	0	0	0.0
No	67	91	73.6
Xanthine inhibitors			
Yes	3	3	100.0

Table 3: Positive Predictive Value of Claims for Asthma, COPD, or Both in the 6 Months Prior to Cohort Entry

	Number of Confirmed Cases	Medical Records Received	Positive Predictive Value
No	64	88	72.7
Prescriber Specialty:			
Family/general provider			
Yes	36	48	75.0
No	31	43	72.1
Allergy/immunology			
Yes	15	20	75.0
No	52	71	73.2
Internal medicine			
Yes	30	42	71.4
No	37	49	75.5
Pulmonology			
Yes	13	17	76.5
No	54	74	73.0
Allied health professional			
Yes	0	0	0.0
No	67	91	73.6
Other			
Yes	12	19	63.2
No	55	72	76.4
Diagnoses:			
Upper respiratory tract infections			
Yes	40	49	81.6
No	27	42	64.3
Lower respiratory tract infection			
Yes	67	91	73.6
No	0	0	0.0
Bronchitis/Bronciolitis			
Yes	19	24	79.2
No	48	67	71.6
Congestive heart failure			
Yes	1	1	100.0
No	66	90	73.3
Other:			
Spirometry procedure			
Yes	26	33	78.8
No	41	58	70.7
Claim for COPD Only			
Overall	75	92	81.5
Age, years			
20-39	2	4	50.0
40-64	44	58	75.9
> 64	29	30	96.7
Sex			
Male	38	42	90.5
Female	37	50	74.0
Top 3 Variables Predictive of COPD Confirmation			
6. 65+ years of age			
Yes	29	30	96.7
No	46	62	74.2
7. Inhaled anticholinergic drug dispensing			
Yes	33	35	94.3
No	42	57	73.7
8. Radiologic examination, chest, 2 views, frontal and lateral (CPT 71020)			
Yes	48	54	88.9
No	27	38	71.1

Table 3: Positive Predictive Value of Claims for Asthma, COPD, or Both in the 6 Months Prior to Cohort Entry

	Number of Confirmed Cases	Medical Records Received	Positive Predictive Value
Covariate Patterns, Top 3 COPD Variables			
Variable 6, Variable 7, Variable 8[1]			
No, No, No	10	19	52.6
No, No, Yes	16	21	76.2
No, Yes, No	6	7	85.7
No, Yes, Yes	14	15	93.3
Yes, No, No	7	8	87.5
Yes, No, Yes	9	9	100.0
Yes, Yes, No	4	4	100.0
Yes, Yes, Yes	9	9	100.0
Pre-defined Covariates			
Medications:			
Previous LABA			
Yes	3	5	60.0
No	72	87	82.8
Previous ICS			
Yes	4	4	100.0
No	71	88	80.7
Inhaled anticholinergics			
Yes	33	35	94.3
No	42	57	73.7
Systemic corticosteroids			
Yes	29	36	80.6
No	46	56	82.1
Leukotriene modifiers			
Yes	5	9	55.6
No	70	83	84.3
Mast cell stabilizers			
Yes	0	1	0.0
No	75	91	82.4
IgE blockers			
Yes	0	0	0.0
No	75	92	81.5
Xanthine inhibitors			
Yes	2	2	100.0
No	73	90	81.1
Prescriber Specialty:			
Family/general provider			
Yes	40	48	83.3
No	35	44	79.6
Allergy/immunology			
Yes	0	3	0.0
No	75	89	84.3
Internal medicine			
Yes	34	42	81.0
No	41	50	82.0
Pulmonology			
Yes	29	32	90.6
No	46	60	76.7
Allied health professional			
Yes	2	2	100.0
No	73	90	81.1
Other			
Yes	16	20	80.0
No	59	72	81.9
Diagnoses:			
Upper respiratory tract infections			
Yes	12	20	60.0

Table 3: Positive Predictive Value of Claims for Asthma, COPD, or Both in the 6 Months Prior to Cohort Entry

	Number of Confirmed Cases	Medical Records Received	Positive Predictive Value
No	63	72	87.5
Lower respiratory tract infection			
Yes	75	92	81.5
No	0	0	0.0
Bronchitis/Bronciolitis			
Yes	51	59	86.4
No	24	33	72.7
Congestive heart failure			
Yes	13	13	100.0
No	62	79	78.5
Other:			
Spirometry procedure			
Yes	32	39	82.1
No	43	53	81.1
Claim for Asthma and COPD (PPV of Asthma Only)			
Overall	40	104	38.5
Age, years			
20-39	5	6	83.3
40-64	30	76	39.5
> 64	5	22	22.7
Sex			
Male	19	44	43.2
Female	21	60	35.0
Top 3 Variables Predictive of Asthma Only Among Patients with Claims-based COPD and Asthma			
11. Chronic airway obstruction, not elsewhere classified (ICD-9 496.xx)			
Yes	20	76	26.3
No	20	28	71.4
12. General Bronchodilator Agents			
Yes	6	35	17.1
No	34	69	49.3
13. Inhaled corticosteroid dispensing			
Yes	9	39	23.1
No	31	65	47.7
Covariate Patterns, Top 3 Asthma and COPD Variables			
Variable 11, Variable 12, Variable 13[1]			
No, No, No	17	23	73.9
No, No, Yes	2	2	100.0
No, Yes, No	0	0	0.0
No, Yes, Yes	1	3	33.3
Yes, No, No	14	42	33.3
Yes, No, Yes	1	2	50.0
Yes, Yes, No	0	0	0.0
Yes, Yes, Yes	5	32	15.6
Pre-defined Covariates			
Medications:			
Previous LABA			
Yes	3	12	25.0
No	37	92	40.2
Previous ICS			
Yes	4	14	28.6
No	36	90	40.0
Inhaled anticholinergics			
Yes	9	39	23.1
No	31	65	47.7
Systemic corticosteroids			
Yes	27	70	38.6
No	13	34	38.2

Table 3: Positive Predictive Value of Claims for Asthma, COPD, or Both in the 6 Months Prior to Cohort Entry

	Number of Confirmed Cases	Medical Records Received	Positive Predictive Value
Leukotriene modifiers			
Yes	10	30	33.3
No	30	74	40.5
Mast cell stabilizers			
Yes	0	0	0.0
No	40	104	38.5
IgE blockers			
Yes	0	0	0.0
No	40	104	38.5
Xanthine inhibitors			
Yes	2	6	33.3
No	38	98	38.8
Prescriber Specialty:			
Family/general provider			
Yes	22	67	32.8
No	18	37	48.7
Allergy/immunology			
Yes	7	10	70.0
No	33	94	35.1
Prescriber Specialty, cont.:			
Internal medicine			
Yes	25	59	42.4
No	15	45	33.3
Pulmonology			
Yes	19	52	36.5
No	21	52	40.4
Allied health professional			
Yes	1	2	50.0
No	39	102	38.2
Other			
Yes	10	21	47.6
No	30	83	36.1
Diagnoses:			
Upper respiratory tract infections			
Yes	20	43	46.5
No	20	61	32.8
Lower respiratory tract infection			
Yes	40	104	38.5
No	0	0	0.0
Bronchitis/Bronciolitis			
Yes	28	72	38.9
No	12	32	37.5
Congestive heart failure			
Yes	3	9	33.3
No	37	95	39.0
Other:			
Spirometry procedure			
Yes	21	53	39.6
No			
No Claims for Asthma or COPD (PPV of Asthma Only)			
Overall	24	83	28.9
Age, years			
20-39	7	17	41.2
40-64	14	57	24.6
> 64	3	9	33.3
Sex			
Male	8	32	25.0
Female	16	51	31.4

Table 3: Positive Predictive Value of Claims for Asthma, COPD, or Both in the 6 Months Prior to Cohort Entry

	Number of Confirmed Cases	Medical Records Received	Positive Predictive Value
Top 3 Variables Predictive of Asthma Only			
16. Lipid panel (CPT 80061) Among Patients with Neither Claims-based COPD nor Asthma			
Yes	1	23	4.4
No	23	60	38.3
17. Collection of venous blood by venipuncture (CPT 36415)			
Yes	4	31	12.9
No	20	52	38.5
18. Macrolides			
Yes	4	29	13.8
No	20	54	37.0
Covariate Patterns, Top 3 Neither Asthma Nor COPD Variables			
Variable 16, Variable 17, Variable 18[1]			
No, No, No	16	33	48.5
No, No, Yes	4	15	26.7
No, Yes, No	3	9	33.3
No, Yes, Yes	0	3	0.0
Yes, No, No	0	2	0.0
Yes, No, Yes	0	2	0.0
Yes, Yes, No	1	10	10.0
Yes, Yes, Yes	0	9	0.0
Pre-defined Covariates			
Medications:			
Previous LABA			
Yes	0	4	0.0
No	24	79	30.4
Previous ICS			
Yes	0	2	0.0
No	24	81	29.6
Inhaled anticholinergics			
Yes	0	2	0.0
No	24	81	29.6
Systemic corticosteroids			
Yes	9	28	32.1
No	15	55	27.3
Leukotriene modifiers			
Yes	5	11	45.5
No	19	72	26.4
Mast cell stabilizers			
Yes	0	0	0.0
No	24	83	28.9
IgE blockers			
Yes	0	0	0.0
No	24	83	28.9
Xanthine inhibitors			
Yes	0	0	0.0
No	24	83	28.9
Prescriber Specialty:			
Family/general provider			
Yes	12	48	25.0
No	12	35	34.3
Allergy/immunology			
Yes	1	1	100.0
No	23	82	28.1
Internal medicine			

Table 3: Positive Predictive Value of Claims for Asthma, COPD, or Both in the 6 Months Prior to Cohort Entry

	Number of Confirmed Cases	Medical Records Received	Positive Predictive Value
Yes	7	28	25.0
No	17	55	30.9
Pulmonology			
Yes	3	7	42.9

Table 3: Positive Predictive Value of Claims for Asthma, COPD, or Both in the 6 Months Prior to Cohort Entry

	Number of Confirmed Cases	Medical Records Received	Positive Predictive Value
No	21	76	27.6
Allied health professional			
Yes	0	0	0.0
No	24	83	28.9
Prescriber Specialty, cont.:			
Other			
Yes	5	25	20.0
No	19	58	32.8
Diagnoses:			
Upper respiratory tract infections			
Yes	5	30	16.7
No	19	53	35.9
Lower respiratory tract infection			
Yes	6	33	18.2
No	18	50	36.0
Bronchitis/Bronchiolitis			
Yes	5	29	17.2
No	19	54	35.2
Congestive heart failure			
Yes	0	1	0.0
No	24	82	29.3
Other:			
Spirometry procedure			
Yes	3	8	37.5
No	21	75	28.0

Abbreviations: COPD, chronic obstructive pulmonary disease; CPT, current procedural terminology

[1]Numbered above. Yes indicates presence of that variable, No indicates absence of that variable.

Table 4: Positive Predictive Value of Claims for Asthma, COPD, or Both in the 12 Months Prior to Cohort Entry

	Number of Confirmed Cases	Medical Records Received	Positive Predictive Value
Claim for Asthma Only			
Overall	55	77	71.4
Age, years			
20-39	15	19	78.9
40-64	37	53	69.8
> 64	3	5	60.0
Sex			
Male	18	27	66.7
Female	37	50	74.0
Top 3 Variables Predictive of Asthma Confirmation			
1. General medical examination (ICD-9 V70)			
Yes	18	18	100.0
No	37	59	62.7
2. Outpatient visit, moderate complexity (CPT 99214)			
Yes	47	59	79.7
No	8	18	44.4
3. General symptoms (ICD-9 780.xx)			
Yes	27	31	87.1
No	28	46	60.9
Covariate Patterns, Top 3 Asthma Variables			
Variable 1, Variable 2, Variable 3[1]			
No, No, No	3	12	25.0
No, No, Yes	2	3	66.7
No, Yes, No	16	25	64.0
No, Yes, Yes	16	19	84.2
Yes, No, No	1	1	100.0
Yes, No, Yes	2	2	100.0
Yes, Yes, No	8	8	100.0
Yes, Yes, Yes	7	7	100.0
Medications:			
Previous LABA			
Yes	5	6	83.3
No	50	71	70.4
Previous ICS			
Yes	8	10	80.0
No	47	67	70.2
Inhaled anticholinergics			
Yes	0	0	0.0
No	55	77	71.4
Systemic corticosteroids			
Yes	35	47	74.5
No	20	30	66.7
Leukotriene modifiers			
Yes	22	28	78.6
No	33	49	67.4
Mast cell stabilizers			
Yes	1	2	50.0
No	54	75	72.0

IgE blockers			
Yes	0	0	0.0
No	55	77	71.4
Xanthine inhibitors			
Yes	3	3	100.0
No	52	74	70.3
Prescriber Specialty:			
Family/general provider			
Yes	34	44	77.3
No	21	33	63.6
Allergy/immunology			
Yes	13	18	72.2
No	42	59	71.2
Internal medicine			
Yes	31	44	70.5
No	24	33	72.7
Pulmonology			
Yes	12	15	80.0
No	43	62	69.4
Allied health professional			
Yes	1	2	50.0
No	54	75	72.0
Other			
Yes	11	16	68.8
No	44	61	72.1
Diagnoses:			
Upper respiratory tract infections			
Yes	38	48	79.2
No	17	29	58.6
Lower respiratory tract infection			
Yes	55	77	71.4
No	0	0	0.0
Bronchitis/Bronciolitis			
Yes	20	28	71.4
No	35	49	71.4
Congestive heart failure			
Yes	1	1	100.0
No	54	76	71.1
Other:			
Spirometry procedure			
Yes	27	35	77.1
No	28	42	66.7
Claim for COPD Only			
Overall	61	78	78.2
Age, years			
20-39	2	4	50.0
40-64	35	49	71.4
> 64	24	25	96.0
Sex			
Male	31	35	88.6
Female	30	43	69.8
Top 3 Variables Predictive of COPD Confirmation			
6. Inhaled anticholinergic drug dispensing			

Yes	30	32	93.8
No	31	46	67.4
7. 65+ years of age			
Yes	24	25	96.0
No	37	53	69.8
8. Rhinitis (ICD-9 477.xx)			
Yes	6	13	46.2
No	55	65	84.6
Covariate Patterns, Top 3 COPD Variables			
Variable 6, Variable 7, Variable 8[1]			
No, No, No	15	22	68.2
No, No, Yes	4	11	36.4
No, Yes, No	11	12	91.7
No, Yes, Yes	1	1	100.0
Yes, No, No	18	20	90.0
Yes, No, Yes	0	0	0.0
Yes, Yes, No	11	11	100.0
Yes, Yes, Yes	1	1	100.0
Pre-defined Covariates			
Medications:			
Previous LABA			
Yes	3	5	60.0
No	58	73	79.5
Previous ICS			
Yes	4	4	100.0
No	57	74	77.0
Inhaled anticholinergics			
Yes	30	32	93.8
No	31	46	67.4
Systemic corticosteroids			
Yes	30	37	81.1
No	31	41	75.6
Leukotriene modifiers			
Yes	6	10	60.0
No	55	68	80.9
Mast cell stabilizers			
Yes	0	1	0.0
No	61	77	79.2
IgE blockers			
Yes	0	0	0.0
No	61	78	78.2
Xanthine inhibitors			
Yes	1	1	100.0
No	60	77	77.9
Prescriber Specialty:			
Family/general provider			
Yes	34	42	81.0
No	27	36	75.0
Allergy/immunology			
Yes	0	4	0.0
No	61	74	82.4
Internal medicine			
Yes	35	44	79.6

No	26	34	76.5
Pulmonology			
Yes	25	29	86.2
No	36	49	73.5
Allied health professional			
Yes	3	3	100.0
No	58	75	77.3
Other			
Yes	13	17	76.5
No	48	61	78.7
Diagnoses:			
Upper respiratory tract infections			
Yes	16	24	66.7
No	45	54	83.3
Lower respiratory tract infection			
Yes	61	78	78.2
No	0	0	0.0
Bronchitis/Bronciolitis			
Yes	46	54	85.2
No	15	24	62.5
Congestive heart failure			
Yes	15	15	100.0
No	46	63	73.0
Other:			
Spirometry procedure			
Yes	26	34	76.5
No	35	44	79.6
Claim for Asthma and COPD (PPV of Asthma Only)			
Overall	35	90	38.9
Age, years			
20-39	4	5	80.0
40-64	26	65	40.0
> 64	5	20	25.0
Sex			
Male	16	36	44.4
Female	19	54	35.2
Top 3 Variables Predictive of Asthma Only Among Patients with Claims-based COPD and Asthma			
11. Chronic airway obstruction, not elsewhere classified (ICD-9 496.xx)			
Yes	18	66	27.3
No	17	24	70.8
12. General Bronchodilator Agents			
Yes	6	32	18.8
No	29	58	50.0
13. Inhaled corticosteroid dispensing			
Yes	9	37	24.3
No	26	53	49.1
Covariate Patterns, Top 3 Asthma and COPD Variables			
Variable 11, Variable 12, Variable 13[1]			
No, No, No	13	18	72.2
No, No, Yes	3	3	100.0
No, Yes, No	0	0	0.0
No, Yes, Yes	1	3	33.3
Yes, No, No	13	35	37.1

Yes, No, Yes	0	2	0.0
Yes, Yes, No	0	0	0.0
Yes, Yes, Yes	5	29	17.2
Pre-defined Covariates			
Medications:			
Previous LABA			
Yes	4	11	36.4
No	31	79	39.2
Previous ICS			
Yes	5	13	38.5
No	30	77	39.0
Inhaled anticholinergics			
Yes	9	37	24.3
No	26	53	49.1
Systemic corticosteroids			
Yes	23	64	35.9
No	12	26	46.2
Leukotriene modifiers			
Yes	10	26	38.5
No	25	64	39.1
Mast cell stabilizers			
Yes	0	0	0.0
No	35	90	38.9
IgE blockers			
Yes	0	0	0.0
No	35	90	38.9
Xanthine inhibitors			
Yes	4	8	50.0
No	31	82	37.8
Prescriber Specialty:			
Family/general provider			
Yes	21	60	35.0
No	14	30	46.7
Allergy/immunology			
Yes	6	9	66.7
No	29	81	35.8
Internal medicine			
Yes	22	52	42.3
No	13	38	34.2
Pulmonology			
Yes	17	48	35.4
No	18	42	42.9
Allied health professional			
Yes	1	4	25.0
No	34	86	39.5
Other			
Yes	9	19	47.4
No	26	71	36.6
Diagnoses:			
Upper respiratory tract infections			
Yes	19	42	45.2
No	16	48	33.3
Lower respiratory tract infection			

Yes	35	90	38.9
No	0	0	0.0
Bronchitis/Bronciolitis			
Yes	25	66	37.9
No	10	24	41.7
Congestive heart failure			
Yes	3	9	33.3
No	32	81	39.5
Other:			
Spirometry procedure			
Yes	18	51	35.3
No	17	39	43.6
No Claims for Asthma or COPD (PPV of Asthma Only)			
Overall	18	65	27.7
Age, years			
20-39	5	13	38.5
40-64	10	43	23.3
> 64	3	9	33.3
Sex			
Male	6	24	25.0
Female	12	41	29.3
Top 3 Variables Predictive of Asthma Only			
16. General symptoms (ICD-9 780.xx)			
Yes	12	30	40.0
No	6	35	17.1
17. Other and unspecified disorders of back (ICD-9 724.xx)			
Yes	7	13	53.9
No	11	52	21.2
18. Urinalysis, by dip stick or tablet reagent (CPT 81001)			
Yes	6	10	60.0
No	12	55	21.8
Covariate Patterns, Top 3 Neither Asthma Nor COPD Variables			
Variable 16, Variable 17, Variable 18[1]			
No, No, No	4	29	13.8
No, No, Yes	0	2	0.0
No, Yes, No	1	3	33.3
No, Yes, Yes	1	1	100.0
Yes, No, No	3	15	20.0
Yes, No, Yes	4	6	66.7
Yes, Yes, No	4	8	50.0
Yes, Yes, Yes	1	1	100.0
Pre-defined Covariates			
Medications:			
Previous LABA			
Yes	1	2	50.0
No	17	63	27.0
Previous ICS			
Yes	1	3	33.3
No	17	62	27.4
Inhaled anticholinergics			
Yes	0	2	0.0
No	18	63	28.6
Systemic corticosteroids			

Yes	8	27	29.6
No	10	38	26.3
Leukotriene modifiers			
Yes	5	11	45.5
No	13	54	24.1
Mast cell stabilizers			
Yes	0	0	0.0
No	18	65	27.7
IgE blockers			
Yes	0	0	0.0
No	18	65	27.7
Xanthine inhibitors			
Yes	0	0	0.0
No	18	65	27.7
Prescriber Specialty:			
Family/general provider			
Yes	10	38	26.3
No	8	27	29.6
Allergy/immunology			
Yes	2	3	66.7
No	16	62	25.8
Internal medicine			
Yes	7	28	25.0
No	11	37	29.7
Pulmonology			
Yes	3	9	33.3
No	15	56	26.8
Allied health professional			
Yes	0	0	0.0
No	18	65	27.7
Other			
Yes	3	18	16.7
No	15	47	31.9
Diagnoses:			
Upper respiratory tract infections			
Yes	8	31	25.8
No	10	34	29.4
Lower respiratory tract infection			
Yes	11	37	29.7
No	7	28	25.0
Bronchitis/Bronchiolitis			
Yes	5	25	20.0
No	13	40	32.5
Congestive heart failure			
Yes	1	3	33.3
No	17	62	27.4
Other:			
Spirometry procedure			
Yes	4	10	40.0
No	14	55	25.5

Abbreviations: COPD, chronic obstructive pulmonary disease; CPT, current procedural terminology

[1]Numbered above. Yes indicates presence of that variable, No indicates absence of that variable.

Figure 1. Reasons for Non-Procurement of Medical Records (N=244)

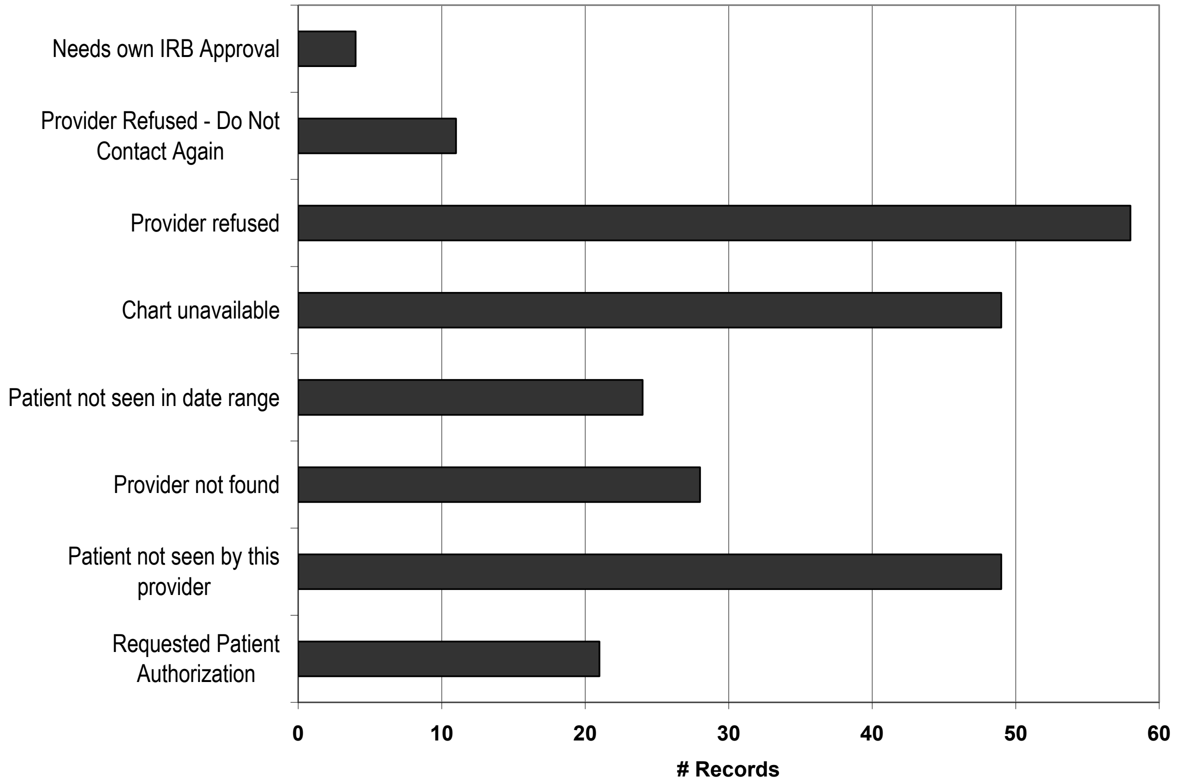

Figure 2. Summary of Asthma and/or COPD Case Identification and Adjudication

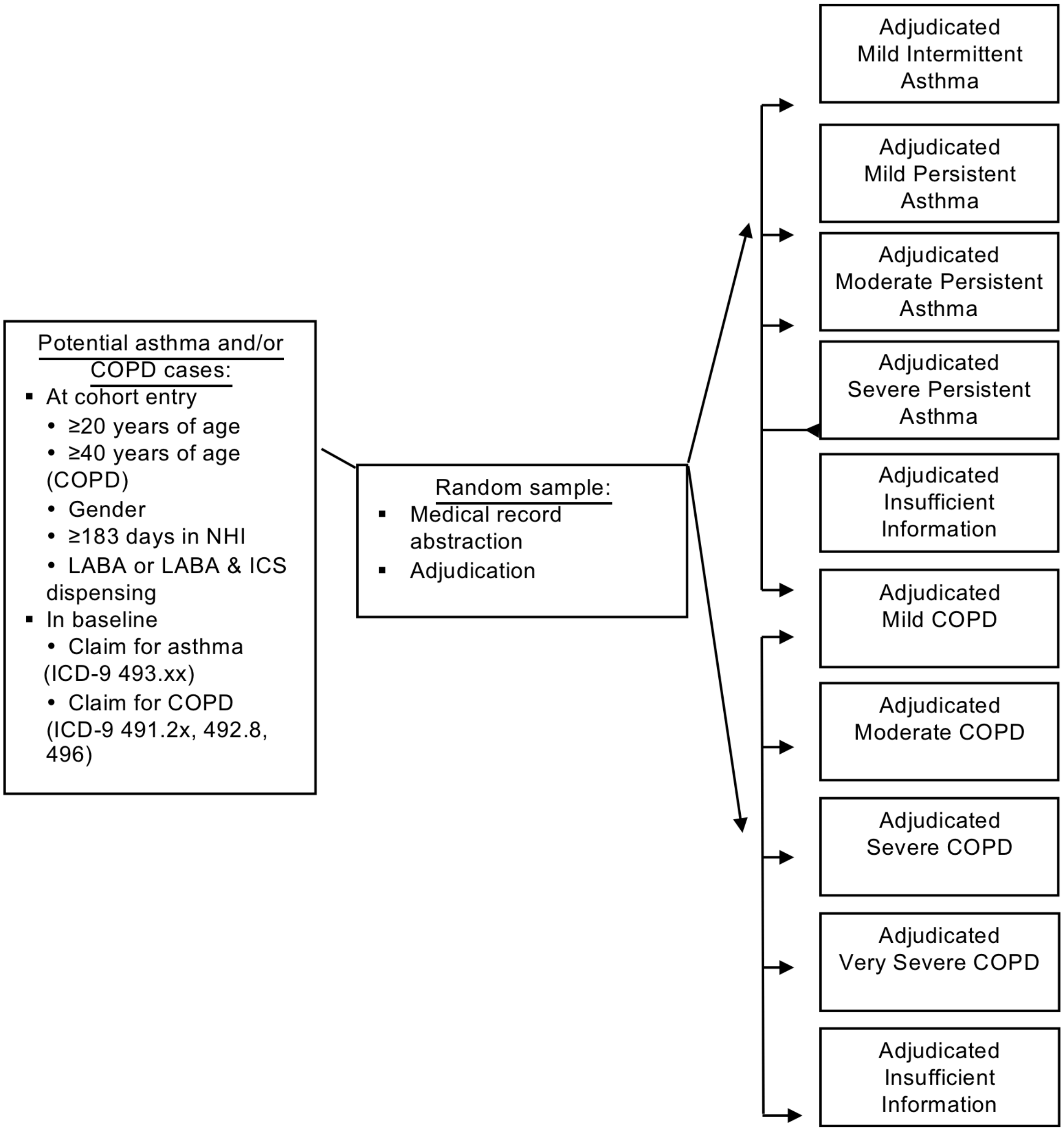

Supplement: Additional file 1 — Identification of Prevalent Asthma and Chronic Obstructive Pulmonary Disease among Initiators of Long-Acting β-Agonists in Health Insurance Claims Data. [file 1471-2466-14-47-S1.pdf]
